# The relationship between health literacy and quality of life among frequent users of health care services: a cross-sectional study

**DOI:** 10.1186/s12955-017-0716-7

**Published:** 2017-07-06

**Authors:** Éva Marjorie Couture, Maud-Christine Chouinard, Martin Fortin, Catherine Hudon

**Affiliations:** 1 Département de médecine de famille et de médecine d’urgence, Université de Sherbrooke, 3001 12e Avenue Nord, Sherbrooke, Québec J1H 5N4 Canada; 2 Département des sciences de la santé, Université du Québec à Chicoutimi, 555, boulevard de l’Université, Saguenay, Québec G7H 2B1 Canada; 3 Centre de recherche du Centre hospitalier universitaire de Sherbrooke, 3001 12e Avenue Nord, Sherbrooke, Québec J1H 5N4 Canada

**Keywords:** Frequent users, Primary care, Health literacy, Quality of life, Chronic disease

## Abstract

**Background:**

Although health literacy and quality of life are important concepts in health care, the link between them is unclear, especially for a population of frequent users of health care services with chronic diseases. Low health literacy is a common problem that has been linked to several negative health outcomes. Quality of life is an important health outcome in patient-centered care. Frequent users of health care services are a vulnerable population that deserves attention due to high costs and negative outcomes such as lower quality of life and higher mortality. The objective of this study was to examine the relationship between health literacy and the physical and mental components of quality of life among frequent users of health care services with chronic diseases.

**Methods:**

This study presents the cross-sectional analysis of data collected through the V1SAGES project, a randomized controlled trial on the effectiveness of a case management intervention in primary care in Quebec, Canada. Participants (*n* = 247) were frequent users of health care services presenting at least one chronic condition. Health literacy was measured by the Newest Vital Sign (NVS), and the physical and mental components of quality of life were evaluated by the Short Form Health Survey Version 2 (SF-12v2). The association between health literacy (independent variable) and the physical and mental components of quality of life was examined using biserial correlation.

**Results:**

No association was found between health literacy and quality of life (physical component: *r* = 0.108, *ρ* = 0.11; mental component: *r* = 0.147, *ρ* = 0.15).

**Conclusion:**

This study suggests that there is no relationship between health literacy and the physical and mental components of quality of life among frequent users of health care services.

**Trial registration:**

NCT01719991. Registered October 25, 2012.

## Background

Health literacy is an emerging concept that has been growing in importance in the last decades [1–3]. Although there is no consensus on the definition; it remains important considering the great prevalence of low health literacy and its impacts on health [4–9]. The World Health Organization (WHO) defines health literacy as: “the cognitive and social skills which determine the motivation and ability of individuals to gain access to, understand and use information in ways which promote and maintain good health” [10].

Low health literacy is highly prevalent in the general population [5], especially in individuals with chronic diseases [8]. Low health literacy can have numerous negative impacts on health and is associated with a decrease in adherence to treatment [6, 9] and in use of preventive services [4, 7], an increase in number of hospitalisations [11, 12] and health system costs [13], poorer health [7, 14] and higher mortality risk [1, 15]. Health literacy could also be a better health indicator than age, income, employment status and education [16].

The relationship between health literacy and quality of life, an important patient-centered outcome often evaluated in studies, [17] is unclear. A study conducted in 2004 in USA (*n* = 249) showed that lower health literacy was associated with poorer quality of life for a population seen at a university-based family teaching clinic [18]. However, various studies on other types of population reported different results. A study by Wang [19] conducted among 913 women living in a rural region of China, showed that a decrease in health literacy was associated to a decrease in quality of life but only for certain ethnic groups. A study by Macabasco-O’Connel [20] also demonstrated that patients suffering from heart failure (*n* = 605) with adequate health literacy had a better quality of life. Song’s [21] study among men with prostate cancer (*n* = 1581), showed an association only with the mental component of quality of life. Finally, a study by Smith [22] conducted in a Canadian population of 259 adults in primary care, did not demonstrate an association between health literacy and quality of life. In light of these different results, it is possible that the relationship between health literacy and quality of life depends on certain aspects: specific chronic diseases, cultural characteristics or aspects of quality of life.

Frequent users of healthcare services may be one of the groups influenced by health literacy. Frequent users can need 80% of resources used while they only count for 10% of the population [23]. They also present more chronic diseases, greater psychological distress, and higher rates of hospitalisation and mortality [24–26]. In this vulnerable population, it was also shown that quality of life may be poorer [27], but no study evaluated the association between health literacy and quality of life. Such an association could prompt the development and implementation of interventions for frequent users of health care services adapted to their level of health literacy [28–30].

The aim of this study was to examine the association between health literacy and the physical and mental components of quality of life among frequent users of health care services with chronic diseases seen in primary care.

## Methods

### Setting and design

This study is a cross-sectional analysis conducted among patients recruited for a larger project, V1SAGES, a randomized controlled trial (RCT) on the effectiveness of a case management intervention by primary care nurses in Family Medicine Groups (FMG) in the Saguenay-Lac-Saint-Jean region of Quebec (Canada) described in a previous article [31]. A FMG is a primary care organization in which a group of family physicians works closely with nurses in the provision of primary care services to a group of registered patients, including patient follow-up, health promotion and preventive care [32].

### Participants

The participants of V1SAGES were identified by their primary care physician using a software program. First, the case management intervention was presented to all primary care physicians working in the four FMGs, for a total of 38 family physicians. Then, a computerized list of their most frequent users of hospital services was delivered to each family physician, namely patients with more than three visits to the emergency and/or hospitalisations in the previous year. This list was obtained using the MAGIC Chronique software (Médiamed Technologies), an information system technology providing support for decision-making to family physicians. From the computerized list, the family physicians could identify eligible patients they believed would benefit the most from the intervention. They could also recommend patients that were not on the list but were considered as complex because they were frequent users of primary care services. Eligible patients had to be aged between 18 and 85 years and affected by at least one chronic disease (diabetes, cardiovascular disease, respiratory disease, musculoskeletal disease and/or chronic pain). Patients with serious cognitive impairment, severe psychiatric illness or a prognosis of less than one year were excluded.

All participants completed and signed an informed consent form. The study was approved by the research ethics board of the Centre intégré universitaire de santé et de services sociaux (CIUSSS) du Saguenay-Lac-Saint-Jean.

### Data collection

Data from participants were collected at baseline (T0), before their randomization to the case management intervention. A self-administered questionnaire was completed in the presence of a research assistant who provided assistance to the participant, ranging from minimal supervision to reading all the questions, if and when needed. Participants with limitations could complete the questionnaire at home in the presence of a research assistant.

Health literacy was measured by the Newest Vital Sign (NVS) [33]. The NVS consists of a nutrition label from an ice cream container presented to the participant who has to answer six questions about it. It takes approximately three (3) minutes to administer. On a range from zero to six, a score of four or higher is considered as adequate health literacy [33]. The NVS is a reliable instrument presenting a Cronbach alpha >0.76 and correlated with the Test of Functional Health Literacy in Adults (TOFHLA), another instrument measuring health literacy (*r* = 0.59, *P* < .001) [33, 34]. The NVS demonstrated high sensitivity and moderate specificity for detecting limited literacy and is a markedly better predictor of patients with low literacy than education or age alone [33]. The Quality of life was measured by the SF-12v2 [35], a short version of the SF-36 [36]. It includes 12 questions and 8 sub-scales, and is used to calculate two component scores, the Physical Component Summary Score (PCS) and the Mental Component Summary Score (MCS), ranging from 0 to 100. Both component summary scores demonstrated high reliability with Cronbach alphas >0.80 and correlated with the EuroQol five dimensions questionnaire (EQ-5D), another instrument measuring quality of life [37]. The Disease Burden Morbidity Assessment (DBMA) was used to evaluate the illness burden of patients. Using a list of 21 conditions, patients rated the degree to which the condition limits his or her daily activities on a five-point descriptive scale [38, 39]. The total score is the sum of the degree of limitation for all conditions. The DBMA was validated in a French-speaking population from Quebec and presented a high test-retest reliability (ICC: 0.86, 95% CI: 0.79–0.92) [39]. The questionnaire also included sociodemographic questions (gender, age, education and family income).

### Sample size

As suggested by Tabachnick and Fidell (2007) [40], the desirable minimum n for testing the significance of individual predictors is *n* > 104 + k (where k is the number of predictable variables in the regression), based on detecting a medium effect size, with α < = .05, and a power of 80%. A minimum sample size of 110 participants was estimated [40]. In the V1SAGES project, 404 patients were identified by the family physicians and contacted to assess eligibility. A total of 247 patients were randomized and were included in this cross-sectional analysis.

### Data analysis

We described the sample using the mean ± standard deviation (SD) for continuous variables and percentage for categorical variables. A biserial correlation between health literacy (independent variable) and the two component scores (PCS and MCS) of quality of life (dependant variable) was performed. Assumptions of biserial correlation were verified and confirmed by a statistician. Health literacy was considered as a dichotomous variable: adequate (NVS ≥ 4) or not (NVS < 4). The two component scores (PCS and MCS) of quality of life were considered as continuous variables. All analyses were performed with PASW Statistics 20 (SPSS Inc.). The α significance level was set at 0.05.

## Results

A total of 247 participants were included in this study (Table 1). Many patients (44.5% male, mean age of 62.8, SD =11.8 years) presented compromised health literacy levels (NVS <4 [33], 67.5%). The mean score for the physical component summary of the SF-12v2 was 37.2, SD = 11.7, and the mean score for the mental component summary was 44.6, SD = 12.2 (Table 2). Most patients presented a high illness burden (DBMA mean 13.4, SD = 8.5) with a mean of six (6) chronic diseases per patient.

**Table 1:** Sample characteristics

Characteristic	Participants	Compromised health literacy (NVS <4), n (%)	QOL (SF-12 V2), Physical component, mean (SD)	QOL (SF-12 V2), Mental component, mean (SD)
Mean age (SD), years	59.9 (13.3)			
Male, n (%)	102 (41.6)			
Illness burden, mean (SD) *1 missing*				
DBMA	13.4 (8.5)			
Education, n (%)				
< 8 years	36 (14.6)	32 (88.9)	37.7 (10.9)	41.5 (11.7)
8–12 years	123 (49.8)	91 (74.0)	36.3 (11.8)	44.8 (12.4)
Professional/trade school	11 (4.4)	7 (63.6)	36.1 (14.8)	41.8 (12.4)
College	52 (21.1)	22 (42.3)	38.7 (12.5)	45.3 (12.0)
University	25 (10.1)	12 (48.0)	38.5 (9.8)	48.1 (11.1)
Family income in CAD, n (%) *5 missing*				
< 10,000$	24 (9.9)	20 (83.3)	35.5 (10.7)	37.3 (14.1)
10,000 to 29,999$	79 (32.7)	63 (79.7)	36.6 (12.1)	45.1 (12.8)
30,000 to 49,999$	75 (31.0)	47 (62.7)	36.5 (12.0)	45.5 (10.8)
≥ 50,000$	64 (26.4)	29 (45.3)	39.3 (11.3)	47.0 (10.7)

*CAD* Canadian dollars, *NVS* Newest Vital Sign, *SF-12v2* Short Form Health Survey, *DBMA* Disease Burden Morbidity Assessment, *SD* standard deviation

**Table 2:** Patient health literacy levels and PCS and MCS for quality of life

Variable	Participants
Health literacy (NVS), n (%) *4 missing*	
NVS < 4	164 (67.5)
NVS ≥ 4	79 (32.5)
QOL (SF-12 V2), mean (SD)	
Physical component	37.2 (11.7)
Mental component	44.6 (12.2)

*NVS* Newest Vital Sign, *SF-12v2* Short Form Health Survey, *SD* standard deviation

In the biserial correlations (Table 3), no association between health literacy and quality of life for the physical component nor the mental component was shown.

**Table 3:** Biserial correlations with Newest Vital Sign

	Correlation coefficient	*Ρ* value
SF-12 V2		
Physical component	0.108	0.11
Mental component	0.147	0.15

## Discussion

This is the first study examining the association between health literacy and physical and mental components of quality of life in frequent users of health care services with chronic diseases in primary care. Results show the absence of an association between these variables. As mentioned previously, the studies evaluating the association between health literacy and quality of life produced mixed results [18–22]. Populations were diverse in terms of culture and geographic location. No other study targeted frequent users of health care services. Only one study was conducted in primary care and did not show a link between these two variables either [22]. Because the two studies conducted in Canada did not show an association, consideration must be given to the impact of the geographical and cultural situation of participants as a potential explanation for part of the results. It is recognized that culture/geographical situation may have an impact on quality of life as it is, in part at least, culturally constructed [27].

No other study used the NVS. Health literacy is not a concept that is easily assessed [41] and no tool commands universal agreement [42]. The other studies cited above used a “homemade” questionnaire [19], the TOFHLA [20], the Rapid Estimate of Adult Literacy in Medicine (REALM) or its adaptations [21, 22]. The TOFHLA, REALM (and its adaptations) and the NVS are measurement tools recognized and validated for health literacy [33, 43–45]. Therefore it is unlikely that the different tools used to measure health literacy, alone, were able to explain an absence of association.

In respect to the measurement of quality of life, only one other study used the SF-12v2 [21]. This study, among 1581 participants with a prostate cancer diagnosis, showed a link between health literacy (measured by the REALM) and the mental health component of quality of life. Each of the other studies mentioned above used a different measure of quality of life, such as the EQ-5D [19], the Heart Failure Symptom Scale (HFSS) [20], the COOP/WONCA Charts [22] or the Healthy Days Core Module (CDC-HRQOL-4) [18]. These tools represents the two main type of approach for measuring quality of life: specific instrument focussing on problems associated with a single disease, patient type, or function; and generic instruments providing health profile applicable for broader type of situation [46].

The SF-36 and its short version, the SF-12, used in this study, are generic tools for the assessment of quality of life [47–49]. Although they are among the most frequently used, no one tool is recognized as the accepted standard to measure quality of life [49]. Again, it would be surprising that the different types of tools used to measure quality of life be able to alone explain the absence of association, especially considering that the presence or absence of an association in the studies cited above, do not seem to depend on the tool used.

This is the first research evaluating the relationship between health literacy and physical and mental components of quality of life among frequent users of health care services. The size of the sample for this type of analysis allows for an adequate statistical power. The study also has its limits. It relied on the secondary analysis of data from the V1SAGES project. The questionnaires were self-administered which may have caused a social desirability bias. Participants may have responded more positively to the questionnaires than what is the real reflection of their situation and thus potentially contribute to results which are partially inaccurate.

Finally, using the NVS to measure health literacy may have resulted in biases. It does not allow us to examine all spheres of health literacy, but relies more on reading skill, reading comprehension and numeracy and its specificity may lead to overestimate the rate of low health literacy [33]. However, it is a recognized tool for health literacy [41, 50] and presents an adequate correlation with the TOFHLA [33]. Health literacy remains a concept that is difficult to properly assess and represents a challenge [41]. The NVS was chosen in this study because of its availability in French-language, takes little time to complete while presenting adequate metrological qualities. A future study using more than one measure of health literacy could be considered. It would then be able to use the NVS, a validated French-language version of the TOFHLA and/or another tool for the measure of health literacy concurrently.

The absence of a relationship between health literacy and quality of life in this study does not mean that we should not address the impact of these variables one on the other, but rather that we should attempt to gain a better understanding and evaluate them in order to get an in-depth comprehension of their mutual interrelations. Nevertheless, as described previously, quality of life and health literacy are linked to the global health of individuals.

In future research it would be interesting to verify if there is an association between these two variables in frequent users of health care services in general. The definition of frequent user is not clearly defined and varies from one study to another [51]. Results may vary if the cut-off is higher. Finally, a next step would be to look at variation in time for health literacy and quality of life, as well as the effect on the relationships in the course of interventions targeting frequent users or individuals with low health literacy.

## Conclusion

This study did not show an association between health literacy and the physical and mental components of quality of life among frequent users of health care services with chronic diseases in primary care. More research is necessary to see if there is an association for other groups of patients, very frequent users of health care services as well as frequent users not necessarily presenting chronic diseases.

## Abbreviations

CAD: Canadian dollars
CIUSSS: Centre intégré universitaire de santé et services sociaux
DBMA: Disease burden morbidity assessment
EQ-5D: EuroQol five dimensions questionnaire
ER: Emergency room
FMG: Family medicine group
HFSS: Heart failure symptom scale
HRQoL: Health related quality of life
MCS: Mental component summary score
NAAL: National assessment of adult literacy
NALS: National adult literacy survey
NVS: Newest vital sign
OHIP: Oral health impact profile
PCS: Physical component summary score
RCT: Randomized controlled trial
REALM: Rapid estimate of adult literacy in medicine
SD: Standard deviation
SF-12 V2: Short form health survey version 2
TOFHLA: Test of functional health literacy in adults
WHO: World Health Organization
